# Supplementary material for: Towards Best-Practice Healthcare for Transgender Patients: Quality Improvement in United Kingdom General Practice
Source: Healthcare (Basel). 2025 Feb 7;13(4):353. doi: 10.3390/healthcare13040353 (PMC11855766; doi:10.3390/healthcare13040353)
Supplement: Supplementary file 1 [file healthcare-13-00353-s001.zip › healthcare-3397933-supplementary.pdf]

healthcare

MDPI

Supplementary Materials S1

Screening for gender variant codes consisted of four stages:

Stage 1: 'Basic search' using codes associated with transgender or transsexual presentations, on or before the defined study date (figure S1).

CTV description	Code	CTV description	Code
Operation for sexual transformation	7L0B.	Female-to-male transsexual	Y1bd5
Other specified operation for sexual transformation	7L0By	Male-to-female transsexual	Y1bd6
Operation for sexual transformation NOS	7L0Bz	Previously bisexual transexual	Y1bd7
Transsexualism	E225.	Non-binary gender	Y1bd8
Transsexuality with unspecified sexual history	E2250	Surgically transgendered transsexual	Y1bd9
Transexuality with asexual history	E2252	Female to male transsexual person on hormone therapy	Y1c27
Transexuality with homosexual history	E2252	Surgically trangendered transexual, female-to-male	Y1c28
Transexuality with heterosexual history	E2253	Male to female transsexual person on hormone therapy	Y1c29
Transsexualism NOS	E225z	female	Y1c2a
[X]Gender identity disorder	Eu64.	Genderfluid	Y1f4d
Gender identity disorder of childhood	Eu642	Transgender female	Y1f4e
[X]Other gender identity disorders	Eu64y	Transgender Male	Y1f4f
[X]Gender identity disorder, unspecified	Eu64z	Transgender	Y1f50
Transsexual	Ua1b8	Identified as female gender	Y1fcd
Transsexual voice	Ub1SQ	Bilateral mastectomy for female to male transsexual	Y2b5c
Gender identity disorder	X00TG	Gender confirmation bottom surgery	Y2b5d
Sexual or gender identity uncertain	X768E	Gender conformation top surgery	Y2b5e
Gender dysphoria	XSEDT	Referral to gender identity clinic	Y379a
Gender reassignment patient	Xa22w	Persons gender identity is not the same as their gender assigned at birth	Y3d13
Transgender identity	Xafge	Identifies as male gender	Y3da0
Intends to or has undertaken thoughts or actions to change birth sex to self-identified gender	Y15a3	Provision of information about NHS population screening programmes available to transgender and non-binary people	Y3da1
		Identifies as non-binary	Y3da2

Figure S1. Basic search

Stage 2: Screening for oestrogen as gender affirming therapy. Search included all patients taking oestrogenic medications (MtF1), then excluded all of those taking a progesterone or with a Mirena in situ (MtF2), then excluding all codes associated only with birth-registered females (MtF4).

Search name	***Search criteria***			
**MtF1**	**Oestrogen oral and transdermal preparations - Exact Drugs search**			
	Estraderm MX 100 patches		Evorel 50 patches	
	Estraderm MX 25 patches		Evorel 75 patches	
	Estraderm MX50 patches		FemSeven 100 patches	
	Estraderm MX 75 patches		FemSeven 50 patches	
	Estradiol 0.06% gel		FemSeven 75 patches	
	Estradiol 1.53mg/dose transdermal spray		Lenzetto 1.53mg/dose transdermal spray	
	Estradiol 1mg gel sachets		Oestrogen pump-pack 0.06% gel	
	Estradiol 1mg tablets		Progynova 1mg tablets	
	Estradiol 2mg tablets		Progynova 2mg tablets	
	Estradiol 500microgram gel sachets		Progynova TS 100micrograms/24hours patches	
	Estradiol valerate 1mg tablets		Progynova TS 50micrograms/24hours patches	
	Estradiol valerate 2mg tablets		Sandrena 1mg gel sachets	
	Estradot 100micrograms/24hours patches		Sandrena 500 microgram gel sachets	
	Estradot 25micrograms/24hours patches		Zumenon 1mg tablets	
	Estradot 75micrograms/24hours patches		Zumenon 2mg tablets	
	Evorel 100 atches		Elleste Solo 1mg tablets	
	Evorel 25 patches		Elleste Solo 2mg tablets	
**MtF2**	**All people with history of taking progesterone (as part of HRT for menopause, or contraception)**			
	Mirena 20 micrograms/24hours intrauterine device		Nexplanon 68mg impnat	
	Progesterone micronised capsules		Etonogestrel 68mg implant	
	Utrogestan 100mg capsules		Desogestrel 75microgram tablets	
**MtF3**	**Results from MtF1, excluding MtF2 (ie on estrogen but not progesterone)**			
**MtF4**	**All patients who have had female-specific conditions coded: hysterectomy, mennorrhagia or menopause, pregnancy, colposcopy**			
	**CTV Description**	**CTV code**	**CTV Description**	**CTV code**
	Menopause	1512	Abdominal hysterectomy	XE06Y
	Menopause symptoms present	66U3.	Vaginal hysterectomy	XE06b
	Menorrhagia	K5920	Menopausal symptoms	XM0t2
	Female climacteric state	K5A2.	Patient pregnant NOS	621Z.
	[X]Other specified menopausal and perim	Kyu9F	Patient currently pregnant	621..
	Hysterectomy	X403B	US female genital system	X70mS
	Premature menopause	X408w	Termination of pregnancy	Xa36H
	Perimenopausal disorder	XE0ev	Female genital and obstetric disorder	X406F
	Perimenopausal menorrhagia	Xa9CP	Colposcopy	XM13i
			Patient currently pregnant	621..
**MtF5**	**Results from MtF3, excluding MtF4 (ie on oestrogren but not progesterone or having had a female-specific condition)**			

Figure S2. Using oestrogen therapy as a search tool for patients on feminising hormones.

Stage 3: Screening for testosterone as gender affirming therapy. Search included all patients taking testosterone-containg medications (FtM1), then excluded all of those with a condition associated only with birth-registered males, or hypogonadism as a male indication for supplemental testosterone (FtM2), then excluding all patients taking testosterone for a menopausal indication (MtF1). See figure S3.

Search name	***Search Criteria***			
**FtM1**	**All patients taking testosterone preparation**			
	Testosterone 16.2mg/g gel			
	Testosterone 2% gel			
	Testosterone 20mg/g transdermal gel			
	Testosterone 40.5mg/2.5g gel unit dose sachets			
	Testosterone 50mg/5g gel unit dose sachets			
	Testisterone 50mg/5g gel unit dose tube			
	Testosteone enantate 250mg/1ml solution for injection ampoules			
	Testosterone undecanoate 1g/4ml solution for injection vials			
	Nebido 1000,g/4ml solution for injection vials			
	Sustanon 250mg/1ml solution for injection ampules			
**FtM2**	**All patients with codes for male-only conditions**			
	**CTV Description**	**CTV code**	**CTV Description**	**CTV code**
	Hypogonadism	X40Ov	Carcinoma in situ of prostate	B834.
	Acquired testicular failure	X401Z	Prostatic disorders NOS	K22z.
	Postirradiation testicular function	C1711	Revision of transurethral prostatecto	X30FK
	Testicular dysfuntion	C17..	Needle biopsy of prostate	X30FR
	Testicular tumour	X400q	Disorder of disorder	X30Oh
	Klinefelter syndrome	PJ7..	Tumour of prostate	X30Ot
	Open prostatectomy	7B36.	Carcinoma of prostate	X78Y6
	Radical prostatectomy with pelvic lym	7B376	Benign prostatic hyperplasia	XE0e6
	Transurethral prostatectomy	7B390	Prostatectomy	XM0o5
	Transurethral laser prostatectomy	7B393	Prostate enlared on PR	XM1N5
	Transrectal needle biopsy of prostate	7B3C2	Biopsy of prostate	XS7LZ
	Malignant tumour of prostate	B46..	Radical prostatectomy	Xa8P6
	Testicular lump	Xa04P	Metastasis from malignant tumour of	XaFrk
	Delayed puberty	X40O7	Urological enlarged prostate	Y3091
	Erectile dysfunction	E2273	Mass of testis	XaBtd
**FtM3**	**FtM1 excluding FtM2 (i.e. all patients on testosterone that do not have a male-only condition)**			
**FtM4**	**FtM3 excluding MtF1 (i.e. all patients on testosterone that do not have a male only condition**			
	**and are not taking oestrogen as well as part of HRT)**			

Figure S3. Using testosterone therapy as a search tool for patients on feminising hormones.

Stage 4: All records in the final patient list manually checked.
